# Point-of-care ultrasound in hemodynamically unstable pulmonary embolism

**DOI:** 10.1186/s13054-026-06097-4

**Published:** 2026-05-21

**Authors:** Oscar Moreno-Loaiza, Cristiano Bandeira de Melo, Milagros Moreno-Loaiza, Simão Bamberg

**Affiliations:** 1 Hospital Estadual Alberto Torres. Secretaria Estadual de Saúde do Rio de Janeiro, Rio de Janeiro, Brazil; 2 Hospital Edgardo Rebagliati Martins, Essalud, Lima, Peru

## Abstract

The recent guideline from the American Heart Association on acute pulmonary embolism provides an updated framework for diagnosis, risk stratification, and management. Computed tomography pulmonary angiography remains the reference standard for confirming pulmonary embolism. However, in critically ill patients with hemodynamic or respiratory instability, transport for definitive imaging may be unsafe or impractical. In this context, bedside evaluation becomes central to early decision-making. Point-of-care ultrasound (POCUS), particularly when performed as a multi-organ assessment integrating cardiac, pulmonary, and venous ultrasound, can rapidly identify signs of right ventricular dysfunction, detect thrombus in transit, and exclude alternative life-threatening causes of shock. Emerging evidence suggests that multi-organ POCUS has high diagnostic performance in critically ill patients with suspected pulmonary embolism. In unstable patients, bedside ultrasound may therefore provide clinically actionable information to support timely therapeutic decisions when definitive imaging is not immediately feasible.

The recent American Heart Association (AHA) guideline on acute pulmonary embolism (PE) provide an updated evidence-based framework for the diagnosis, risk stratification, and management of adults with PE [1]. A major advance is the introduction of a five-category clinical severity classification, ranging from A to E. This classification aims to better align clinical presentation with prognostic assessment and therapeutic decision-making. Within this framework, computed tomography pulmonary angiography (CTPA) remains the reference standard for diagnostic confirmation, supported by robust diagnostic performance [2, 3]. Conversely, echocardiography alone is not recommended to confirm or exclude PE because of its limited sensitivity [4]. While this diagnostic hierarchy is appropriate as a general recommendation in patients with PE, its applicability may be limited in patients presenting with hemodynamic and respiratory instability (classified as categories D–E). In these critically ill patients, the role of echocardiography and point-of-care ultrasound (POCUS) in early management deserves further consideration.

The diagnostic approach to acute PE relies on the integration of clinical probability assessment, D-dimer testing, and confirmatory imaging, as reflected in the algorithm proposed in the guideline [1]. However, in critically ill patients, this sequential approach may be difficult to implement. Transport for CTPA may be unsafe or impractical in patients with evolving shock or severe hypoxemia and has been associated with adverse events [5]. In such scenarios, the absence of immediately available confirmatory imaging may delay potentially life-saving interventions in patients at the highest risk of deterioration.

When definitive imaging is not immediately feasible, bedside assessment becomes central to early decision-making. Hemodynamic compromise in patients classified as D-E in the AHA guideline reflects an obstructive shock state driven by acute RV pressure overload, resulting in RV dysfunction. The guideline emphasizes the role of echocardiographic parameters in evaluating RV dysfunction (RV dilation, reduced tricuspid annular plane systolic excursion, and alterations in pulmonary artery Doppler) and acknowledge that POCUS may serve as an alternative when formal echocardiography is not immediately available [1]. However, in contemporary critical care, POCUS is rarely limited to isolated cardiac assessment. A multi-organ approach integrating cardiac, pulmonary, and venous ultrasound allows clinicians to explore the etiology of shock, with particularly better diagnostic performance in obstructive shock syndrome patients [6, 7]. In addition, POCUS can rapidly exclude alternative life-threatening causes of circulatory and respiratory failure, such as cardiac tamponade or tension pneumothorax [8]. Notably, a recent meta-analysis published after the search period of the AHA guideline reported a pooled sensitivity of 90% (95% CI 0.85–0.94) for multi-organ POCUS in the diagnosis of PE among critically ill patients [9].

Interpretation of POCUS findings should be contextualized with prior imaging whenever available. The presence of pre-existing RV dilation or dysfunction may reduce the diagnostic value of POCUS in PE. A combination of echocardiographic parameters may help differentiate acute from chronic RV involvement: an RV free wall thickness < 5 mm, a tricuspid regurgitation velocity < 3.4 m/s, and the presence of McConnell’s sign may suggest acute RV pressure overload in the appropriate clinical context [10]. Critical care specialists should also be encouraged to perform POCUS because, although rare, direct visualization of thrombi within pulmonary arteries or identification of a thrombus in transit provides diagnostic certainty [8].

Patients in the D and E profiles represent the high-risk spectrum of acute PE, with high mortality rates [11]. In these patients, systemic thrombolysis and anticoagulation reduce mortality when the bleeding risk is acceptable [1]. Clinical deterioration may occur rapidly in this setting, and delays in therapeutic decision-making can be deleterious. In such scenarios, POCUS should not be viewed as a test aimed at establishing diagnostic certainty of PE. Rather, bedside ultrasound may shift the diagnostic probability sufficiently to cross a therapeutic threshold and support a decision about an immediate reperfusion strategy without further testing [12]. Based on these considerations, we propose a simplified POCUS-guided approach that summarizes current recommendations and key findings to support early therapeutic decision-making in unstable patients with suspected PE (Fig. 1).

**Fig. 1:**
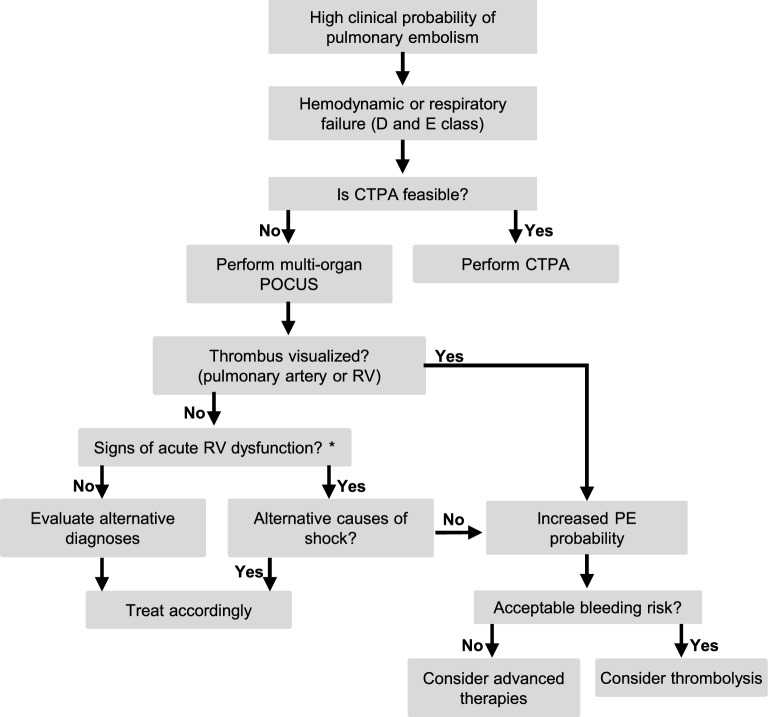
Proposed POCUS-guided decision pathway in hemodynamically unstable patients with suspected pulmonary embolism. * Consider previous imaging when interpreting righ ventricular dysfunction

In selected patients with refractory shock (E2 category), venoarterial extracorporeal membrane oxygenation (VA-ECMO) may be considered as a bridge to reperfusion therapies; and POCUS can facilitate bedside guidance for cannulation in unstable patients [13]. Advanced POCUS parameters may further refine hemodynamic assessment in acute PE. The left ventricle outflow tact velocity–time integral (LVOT—VTI) may identify patients with more severe hemodynamic compromise [14], and could potentially be used to monitor response to therapeutic interventions, such as catheter-directed thrombolyisis or inotropic support [15].

Future research should address whether POCUS-guided decision pathways can safely support therapeutic interventions in hemodynamically unstable patients with suspected PE. Clinical trials evaluating protocols that integrate clinical suspicion with POCUS evidence of RV dysfunction are needed to determine whether such strategies can improve clinical outcomes. In critically ill patients, the key question may not be whether POCUS performs better than CTPA does, but whether bedside ultrasound can provide sufficient diagnostic confidence to justify timely action when definitive imaging is unsafe or unavailable. Such evidence may help refine future recommendations and clarify the role of bedside ultrasound in the early management of unstable patients with PE.

## Data Availability

No datasets were generated or analysed during the current study.

## Abbreviations

AHA: American Heart Association
CTPA: Computed tomography pulmonary angiography
LVOT- VTI: Left ventricle outflow tract velocity–time integral
PE: Pulmonary embolism
POCUS: Point-of-Care Ultrasound
RV: Right Ventricle/Right Ventricular
TAPSE: Tricuspid annular plane systolic excursion
